# Case Report: Respiratory lesions successfully treated with intravenous plasminogen, human-tvmh, replacement therapy in four patients with plasminogen deficiency type 1

**DOI:** 10.3389/fped.2024.1465166

**Published:** 2024-09-20

**Authors:** Charles Nakar, Heather McDaniel, Joseph M. Parker, Karen Thibaudeau, Neelam Thukral, Amy D. Shapiro

**Affiliations:** ^1^ Indiana Hemophilia & Thrombosis Center, Indianapolis, IN, United States; ^2^ Vanderbilt University Medical Center, Nashville, TN, United States; ^3^ Consultant to Kedrion Biopharma Inc., Fort Lee, NJ, United States; ^4^ Global Medical Affairs, Kedrion SpA, Laval, QC, Canada

**Keywords:** airway lesions, case study, hypoplasminogenemia, plasminogen deficiency type 1, plasminogen/human-tvmh, replacement therapy

## Abstract

Plasminogen deficiency type 1 (PLGD-1, hypoplasminogenemia) is an ultra-rare, lifelong disease associated with development of fibrinous lesions in multiple organ systems. Depending on lesion location, clinical manifestations of PLGD-1 can result in acute and/or chronic respiratory airway disease which can compromise respiratory function leading to life-threatening events. Early recognition and effective treatment of airway obstruction caused by fibrinous lesions are critical to prevent morbidity due to respiratory compromise. However, physicians may not be familiar with the clinical presentation and management of PLGD-1, causing delays in diagnosis and treatment and potentially contributing to morbidity. Presented here is a case series of one adult and three pediatric patients with severe respiratory complications of PLGD-1 successfully managed by infusions of plasminogen, human-tvmh replacement therapy. Patients’ respiratory symptoms were resolved or greatly improved, and treatment was generally well tolerated. In all patients, baseline plasminogen activity was substantially increased with plasminogen replacement therapy administered initially every one to two days followed by extended interval dosing as symptoms were controlled or resolved. All four described cases support the clinical benefit of replacement therapy with plasminogen, human-tvmh in the resolution of life-threatening respiratory complications associated with PLGD-1. Clinical manifestations in addition to respiratory lesions were also improved or resolved with continued treatment.

## Introduction

Plasminogen deficiency type 1 (PLGD-1, hypoplasminogenemia) is an ultra-rare disease associated with abnormal accumulation of fibrin-rich lesions on mucous membranes across multiple organ systems, including the respiratory tract (1). PLGD-1 is a lifelong disease; however, the most serious manifestations often occur in infants and young children (2). Plasminogen activity levels (normal 70%–130%, assay dependent) are below 50% in most affected patients (4%–51%, measured in 43 patients with PLGD-1) (1). Ligneous conjunctivitis (LC) is the most common symptom affecting >80% of people with clinical manifestations, while 20%–30% may develop lesions in the respiratory tract (1, 3). Lesions may occur in the tracheobronchial tree and can result in airway compromise and/or obstruction, which can lead to life-threatening events in infants, children, and adults with severe disease (4–7).

In the event of airway compromise/obstruction due to fibrinous lesions, early recognition and effective treatment are key to prevent progression to respiratory failure. As an ultra-rare disease, most physicians are not familiar with the clinical presentation and management of PLGD-1, resulting in delayed diagnosis and treatment. Symptoms suggestive of respiratory involvement include cough, dysphonia, aphonia, stridor, wheezing, and dyspnea (3). The diagnostic odyssey is challenging as disease manifestations vary in terms of age at onset, location, lesion size, and clinical consequences (1, 3, 8). Intravenous plasminogen, human-tvmh (PLG) is the first therapy specifically approved for the treatment of PLGD-1. Prior to the approval of plasminogen replacement therapy, treatment for PLGD-1–associated symptoms consisted of nonspecific medical therapies and surgical interventions with inconsistent and limited success (1, 9–15). Topical plasminogen (eye drops) has shown promise in patients with LC, but is not yet approved for clinical use and is only available for compassionate treatment. Systemic plasminogen replacement therapy is approved as safe and effective for the treatment and prevention of the fibrinous lesions of PLGD-1 with long-term administration (7, 13, 16). Urgent, short-term, or long-term treatment with systemic plasminogen replacement therapy may be needed when treating airway obstruction resulting from fibrinous lesions.

We report a case series of four patients with severe respiratory complications of PLGD-1 successfully managed by infusion of plasminogen, human-tvmh (Kedrion Biopharma).

## Patient 1

At three weeks of age, this patient was diagnosed with PLGD-1 following an upper respiratory infection and development of bilateral LC. Baseline diagnostic characteristics and prior treatments for LC are presented in Table 1. At eight months of age, she received one month of topical treatment with FFP eye drops (initially: 0.5 ml four times daily in the right eye; subsequently: two drops/eye six times daily) and achieved intermittent symptom relief. However, as the LC lesions did not reduce in appearance/size, the treatment was discontinued. At 8.5 months old, she received treatment with investigational plasminogen concentrate ophthalmic drops through a compassionate use protocol with surgical removal of residual fibrinous lesions. Clinically significant improvement in LC was observed and a complete remission was achieved (17).

**Table 1:** Baseline characteristics of patients at time of PLGD-1 diagnosis and prior treatments for LC.

Patient	Age at diagnosis	Age at start of IV PLG	Sex	PLG act at diagnosis (%)^a^	PLG Ag at diagnosis (mg/dl)^b^	Clinical manifestation at diagnosis	Prior treatments for LC
1	3 weeks	16 months	Female	12	–	• Upper respiratory infection • Bilateral LC	• Multiple surgical removals of lesions • Various topical eye drop therapies^c^ (including heparin) • FFP IV infusions and topical eye drops • Topical PLG eye drops (compassionate use)
2	7 months	16 months	Male	<5	–	• Bilateral LC	• Multiple surgical removals of lesions • Various topical eye drop therapies • Topical FFP eye drops
3	6 weeks	3 years	Male	<14	2	• Bilateral LC • Chronic mucopurulent conjunctivitis (L>R)	• Multiple surgical removals of lesions • Various topical eye drop therapies • FFP eye drops (×8/day) with some improvement but required additional 2 surgical removal of lesions within a year (Total ∼2 year therapy with FFP eye drops) • Topical PLG eye drops (compassionate use) ×8/day to left eye, improvement in LC lesions, with residual small, hard lesion
4	14 years	37 years	Female	4	<0.5	• Multiorgan involvement (some of which were debilitating & life-threatening): – Eyes – Nose – Gingiva – Upper airway & lungs – Cervix & uterus – GI tract – Kidney	• Multiple surgical removals of lesions • Various topical eye drop therapies (including heparin and cyclosporine) • IV immunoglobulin • IV FFP • Topical PLG eye drops

Ag, antigen; FFP, fresh frozen plasma; GI, gastrointestinal; LC, ligneous conjunctivitis; PLG, plasminogen; PLGD-1, Plasminogen Deficiency Type 1; IV, intravenous.

^a^ Plasminogen activity levels (normal 70%–130%, assay dependent) are below 50% in most affected patients (4%–51%, measured in 43 patients with PLGD-1).

^b^ Normal plasminogen Ag levels are 5–25 mg/dl, assay dependent.

^c^ Topical therapies usually include one or more of the following: steroids, antibiotics, antihistamines, decongestant.

**Figure 1:**
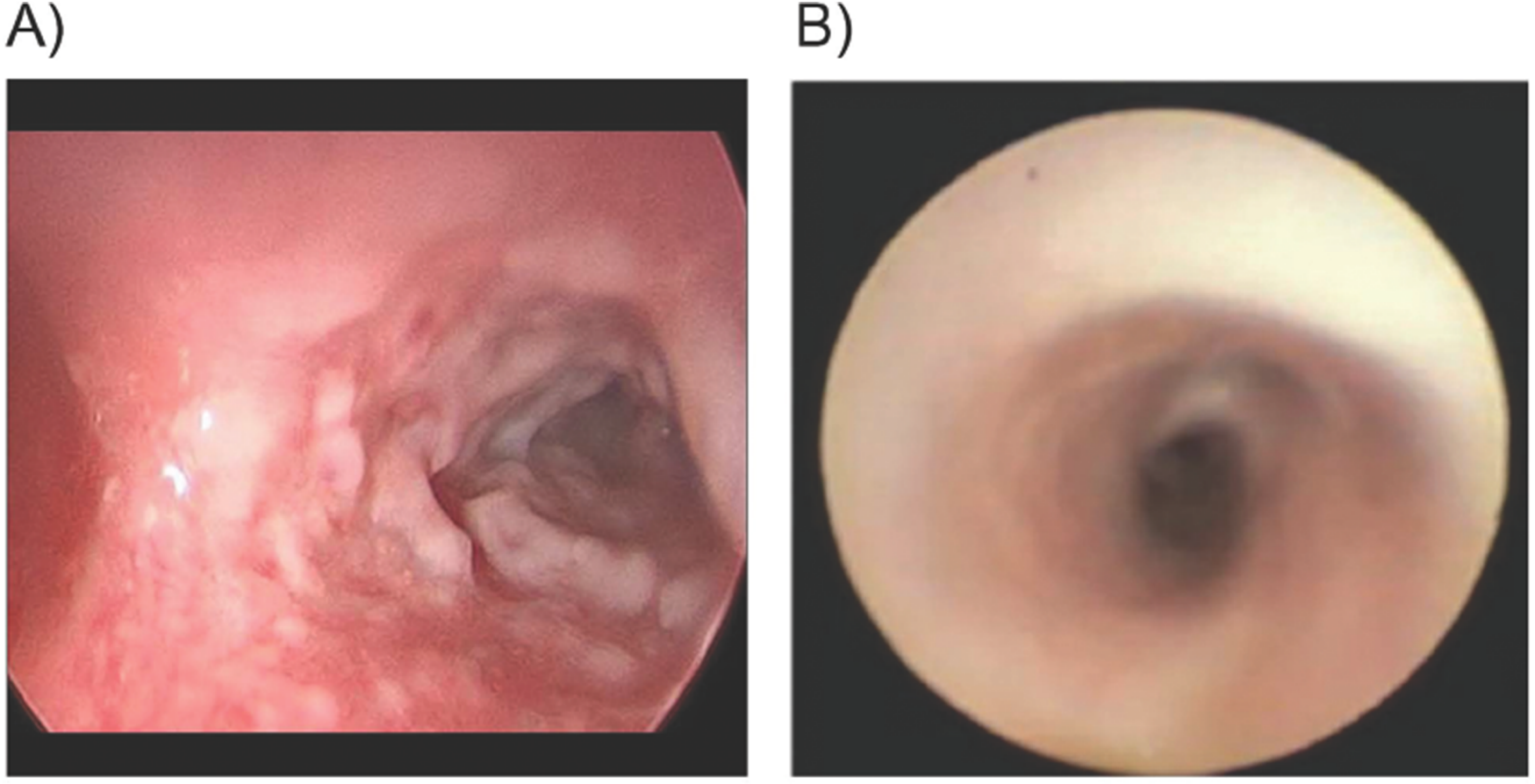
Patient 1. Bronchoscopy of right mainstem bronchus pre-plasminogen, human-tvmh treatment, and 10 days on therapy. **(A)** Carina and right mainstem bronchus: Pre-plasminogen treatment bronchoscopy revealed copious amount of ligneous plaque-like lesions narrowing the airway. **(B)** Carina & right mainstem bronchus: Bronchoscopy on Day 10 of therapy indicates resolution of ligneous lesions.

At 16 months of age, she experienced a febrile illness associated with upper respiratory symptoms and exacerbation of LC with lesion recurrence. Wheezing and stridor progressed to respiratory distress with hypercapnia despite treatment with bronchodilators and corticosteroids (5). Following one dose of IV FFP (15 ml/kg), bronchoscopy revealed friable airways with copious amounts of fibrinous deposits and significant stenosis of the right mainstem bronchus (Figure 1A). Computed tomography (CT) of the chest revealed airway narrowing (R>L) with distal air trapping (5). After admission to the pediatric intensive care unit (PICU) for close monitoring, she received three additional doses of IV FFP (15 ml/kg) every 12 h (5). A peripherally inserted central catheter (PICC) was placed on the second day of admission.

Compassionate use of IV PLG was requested for urgent treatment of life-threatening airway involvement. A 5-day course of prednisolone was initiated on the day before the IV PLG was instituted to reduce airway inflammation. Treatment was initiated in the PICU with PLG 6 mg/kg once daily for the first five days followed by every other day at the same dose/kg. After the first infusion of PLG, plasminogen activity increased from a trough of 27% to a peak value of 107%. Within 48 h of treatment initiation, significant improvement in her respiratory status was observed, with resolution of stridor and tachypnea. Three days after treatment initiation, she was transferred from the PICU to the hematology service for continued treatment and monitoring (5). On Day 10 of PLG treatment, a repeat chest CT scan revealed a patent right bronchus. Repeat bronchoscopy revealed resolution of airway lesions without evidence of airway friability or obstructing membranous airway lesions, and mild to moderate edema in the right and left bronchial trees (Figure 1B). A portacath was placed for outpatient and home therapy simultaneously (Day 10 of therapy). Hospitalization duration was 15 days.

After discharge, she remained on an every other day regimen to complete three weeks of therapy at the same dose. Treatment was modified to three times weekly on Monday-Wednesday-Friday for 5.2 more weeks. As symptoms remained absent, the treatment interval was extended to every three days over a 13.7-week period and then to every four days (for an additional 132 weeks) followed by an every five days regimen. The patient remained symptom-free post-hospitalization and during extension of the dosing intervals (5). The total treatment with PLG was about 268 weeks (5.2 years through April 2022), after which she transitioned to once weekly using the commercially available product.

## Patient 2

**Figure 2:**
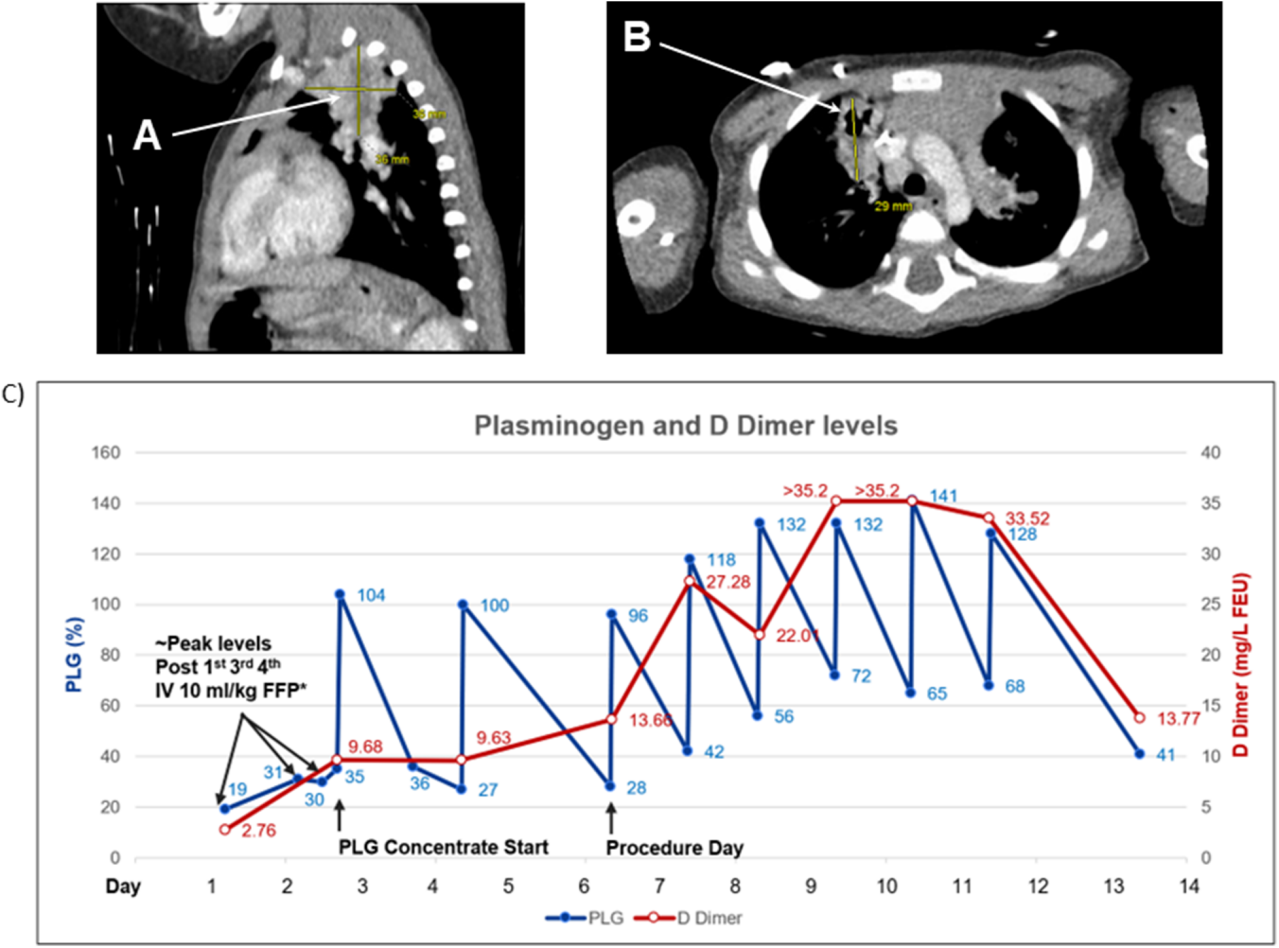
Patient 2. Computed tomography (CT) scan of the chest and plasminogen and D Dimer levels. **(A)** Lobulated heterogeneous soft tissue opacities in upper lobes most likely representing areas of chronic pneumonia. **(B)** Focal areas of bronchiectasis peripherally in the right upper lobe. **(C)** Plasminogen activity level after four infusions of 10 ml/kg fresh frozen plasma (FFP) every 12 h reached a level of 30%–35%. Plasminogen activity after IV 6.6 mg/kg plasminogen, human-tvmh increased on average by 70% (range 60%–76%). D Dimer levels increased with IV FFP (9.68) and with every other day IV 6.6 mg/kg plasminogen, human-tvmh (13.66); significantly increased to a level of >35.2 (maximum level reported by the laboratory) on daily infusions. D Dimer levels signify the fibrinolytic activity of plasminogen and are likely related to the total lesion burden (The D Dimer is less likely related to the procedure type performed). * Plasminogen level drawn within four hours from the end of FFP infusion. PLG, plasminogen.

This patient was diagnosed with PLGD-1 at seven months of age after about four months of eye lesions, confirmed as LC. Baseline diagnostic characteristics and prior treatments for LC are presented in Table 1. His medical history included congenital hydrocephalus (due to Dandy-Walker malformation, which required ventriculoperitoneal shunt with revision due to malfunction), persistent LC in both eyes, recurrent pneumonia and asthma-like symptoms (reported as episodes of status asthmaticus), ascites, and developmental delay (speech and gross motor) (5). At 16 months of age, he was admitted to the PICU for acute-on-chronic lung disease, pneumonia, worsening hoarseness, and extensive LC lesions (Figures 2A, B). Upon admission, FFP transfusions were initiated at 10 ml/kg every 12 h. These interventions provided minimal symptom relief and increases in plasminogen activity with measured values of 30%–35% (5). While on IV FFP treatment, short periods of mild hypoxemia (SpO_2_ 88%) and wheezing were managed with supplemental oxygen via nasal canula and bronchodilators.

Compassionate use IV PLG was requested due to life-threatening airway involvement. PLG infusions (6.6 mg/kg) were initiated on the third day of hospitalization with every other day dosing interval. Plasminogen activity level increased from 35%–104% after the first infusion. Shortly after initiation of PLG, a PICC was placed, and significant increases in D-dimer were documented secondary to the fibrinolytic activity of PLG in the presence of a substantial number of ligneous lesions [peak >35.2 mg/L fibrinogen-equivalent units (FEU)] (Figure 2C). A CT scan revealed chronic lung disease with findings suggestive of fibrinous lesions and evidence of bronchiectasis peripherally in the right upper lobe (5). Lobulated, heterogeneous soft-tissue opacities were observed in both upper lobes (L>R), suggestive of chronic pneumonia. Debris was observed in the left mainstem bronchus, and branching soft-tissue tubular opacities were observed in the left upper lobe, suggestive of plugging in areas of bronchiectasis. Ascites in the upper abdomen was felt to be secondary to the ventriculoperitoneal shunt with poor fluid absorption (5).

Following the third dose of PLG, on the seventh hospitalization day, he underwent rigid and flexible bronchoscopy under general anesthesia and was observed to have ligneous lesions in both main bronchi, as well as middle ear effusions and swelling of the vocal cords. Large ophthalmologic membranes were excised and a portacath was placed for long-term treatment (5). Post-procedure, he was maintained on daily infusions of PLG until post-operative Day 5. Plasminogen peak levels and ∼24-hour trough levels ranged from 96% to 141% and 42% to 72%, respectively. He responded well to therapy with improvement in respiratory status, resolution of tachypnea, hypoxemia and wheezing, improved hoarseness, and no recurrence of eye lesions, and was discharged after 11 days (eight days after starting PLG). On post-operative Day 5, infusions were spaced to every other day for three weeks and weaned to every three to four days.

Subsequent long-term treatment with PLG resulted in improvement of chronic respiratory lesions without exacerbations, no recurrence of LC, and a documented improvement in the patient's developmental delays (5). The cerebrospinal fluid ascites, attributed to plasminogen deficiency and ventriculoperitoneal shunt catheter in the peritoneum, resolved with continued treatment.

His total duration of PLG treatment was approximately 207 weeks (3.9 years through March 2022), after which he was transitioned to the commercially available product administered every fourth day.

## Patient 3

This patient had a history of bilateral LC at about six weeks of age. Baseline diagnostic characteristics and prior treatments for LC are presented in Table 1. After nearly two years of topical FFP therapy, compassionate use of plasminogen ophthalmologic drops was initiated (application eight times daily to left eye) resulting in improvement of LC lesions, although with residual small, hard mucosal membranes. Over the ensuing six months, his parents reported an increase in epiphora and photophobia. Eye discharge was noted in the morning which resolved with application of topical plasminogen eye drops eight times per day in each eye. At age three, he developed an upper respiratory infection with cough and persistent hoarseness over three weeks. Flexible small-scope examination revealed a mass on the right vocal cord and a whitish papillomatous-like mass below the vocal cords consistent with ligneous lesions. The persistence of LC and the new upper airway lesions with the risk of progression to airway compromise and aphonia led the treating physician to request compassionate use of PLG (5).

Treatment with PLG was initiated; 6.6 mg/kg daily for three days, followed by every other day for five doses and every three days for five doses to complete four weeks of therapy. The regimen was modified to every four days for 2.5 months, every five days for one year, followed by a long-term weekly infusion regimen. Documented baseline plasminogen activity level before the first dose of PLG was 12%; corresponding trough plasminogen activity levels measured before subsequent PLG infusions were >10% above baseline levels.

Within four weeks of initiating PLG therapy, hoarseness resolved with significant improvement in his LC. No further clinical respiratory symptoms or LC exacerbations were reported during PLG therapy (5). His total PLG treatment duration was about 122 weeks (2.3 years through March 2022), after which he was transitioned to the commercially available product.

## Patient 4

This patient was diagnosed with PLGD-1 at age 14 years. Her diagnosis was confirmed via genetic testing (15). Heterozygous, single amino acid deletion (c.687_689delGAA, p.Lys230del) in exon 7 of *PLG*, heterozygous frameshift deletion (c.2125_2125delG, p.Gly709fs) in exon 17 of PLG, with 5 heterozygous silent variants (c.330C>T, c.771T>C, c.942C>T, c.1083A>G, and c.2286T>G) and 1 common heterozygous missense variant (c.1414G>A, p.Asp472Asn). Baseline diagnostic characteristics and prior treatments for LC are presented in Table 1. Persistent tracheobronchial lesions were noted at age 15 years and associated with respiratory distress, hypoxemia, and upper airway obstruction for more than two decades requiring numerous surgeries for removal of tracheal, bronchial, and nasal ligneous membranes. She suffered from recurrent right upper lobe collapse and severe bronchiectasis.

**Figure 3:**
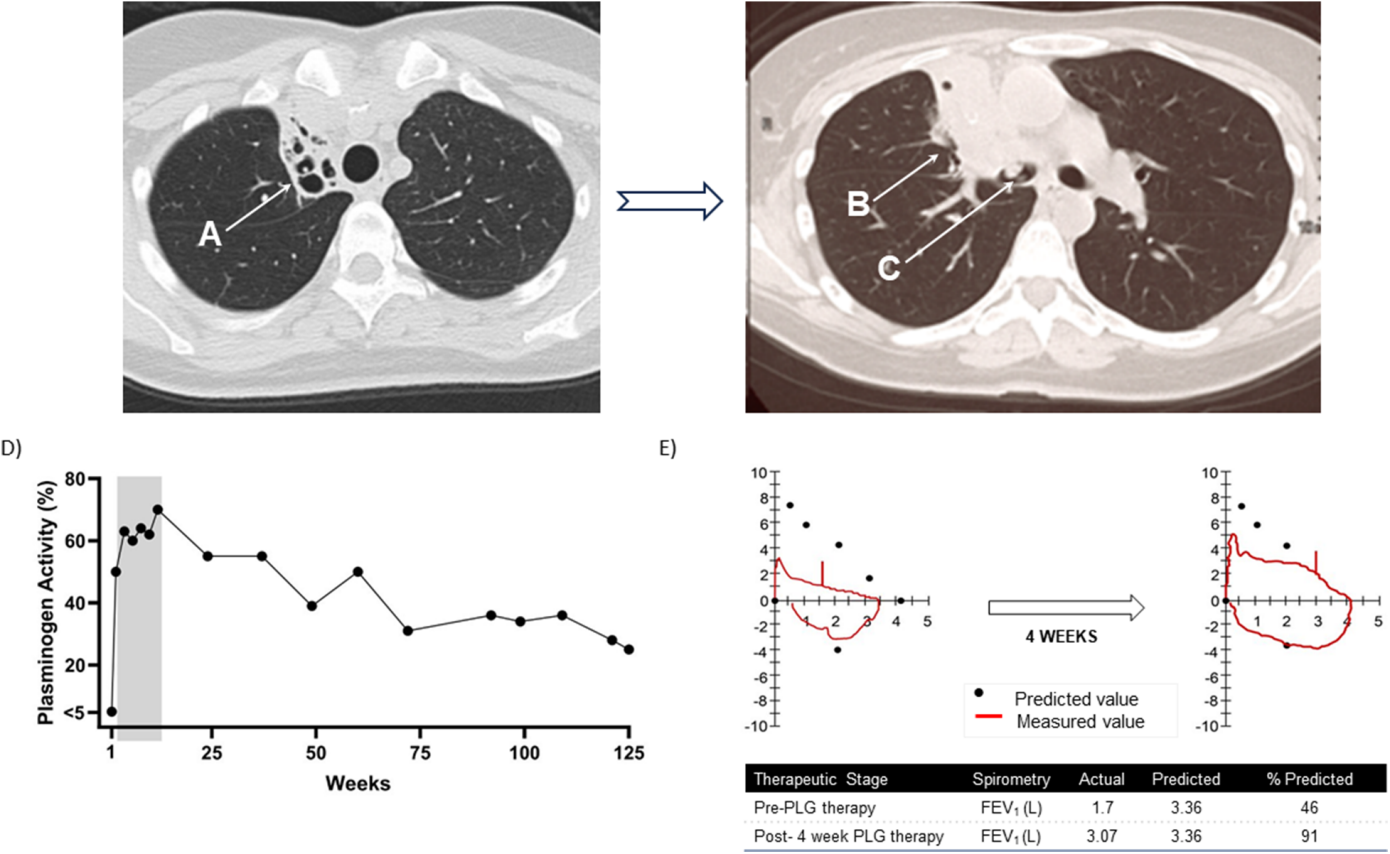
Patient 4. Progression of lung disease on computed tomography (CT) scan of the chest prior to initiating plasminogen, human-tvmh therapy and plasminogen activity and spirometry results at baseline and after four weeks of therapy. **(A)** Progression of right upper lobe atelectasis and bronchiectasis. Chronic right upper lobe atelectasis with severe bronchiectasis. **(B)** Progression to permanent consolidation of the right upper lobe. **(C)** Ligneous lesion in the bronchus intermedius. **(D)** Trough plasminogen activity over 125-week course of plasminogen, human-tvmh therapy. The grey area highlights the increase in plasminogen levels in the first 12 weeks. **(E)** Spirometry results at baseline and after four weeks of therapy indicating significant improvement in spirometry pattern and increase in forced expiratory volume in 1 s (FEV_1_) from 46% to 91% of predicted value.

At the time of entry into the PLG phase 2/3 clinical trial, she had persistent obstructive airway disease with dyspnea. She reported chronic cough with post tussive emesis, chronic use of bronchodilators, inability to exercise or sleep through the night, and impaired quality of life. Her forced expiratory volume in 1 s (FEV_1_) was 1.57 L (46.7% of predicted normal value). A chest radiograph revealed bronchial obstruction with right upper lobe collapse (Figures 3A–C). Three lesions were observed on her bronchus (by bronchoscopy), nasal area (right naris at the inferior turbinate, by otoscope), and renal area (ill-defined bilateral hyperechoic renal lesions, by retroperitoneal ultrasound); other lesions affecting the eyes (LC) and gingiva were noted. She began therapy with PLG 6.6 mg/kg every two days with subsequent modification to every three- or four-day infusions. During the first 12 weeks of PLG therapy, her plasminogen activity levels before infusions (trough) reached the investigational study target threshold (an absolute increase of ≥10% above her baseline value of <5%) at all time points evaluated (Figure 3D) (7, 15).

At the first on-treatment lesion assessment (Week 4), her eye and gingival lesions were resolved, and her bronchial lesion resolved at Week 12. No new or recurrent lesions were reported. Spirometry results over the first 12 weeks of PLG therapy indicated clinically significant improvements from baseline FEV_1_ of 1.57 L (46.7% predicted normal) to 3.07 L (91.4% of predicted normal) at four weeks and maintained at 3.00 L (89.3% of predicted normal) at 12 weeks. Flow volume loops demonstrated variable intrathoracic obstruction which improved from baseline (Figure 3E). At Week 12, a chest CT revealed bronchial lesion improvement with persistent right upper lobe collapse, and a retroperitoneal ultrasound did not show the bilateral hyperechoic lesions previously noted at baseline; otoscope assessment indicated complete resolution of the naris lesion. Her Clinical Global Impression-Global Improvement was much improved through Week 48 of PLG treatment, and her quality-of-life scores improved from 7 (baseline) to 10 at Weeks 8, 12, and 48. Her chronic cough and post tussive emesis were reported as resolved, and she stated she could sleep through the night and resume exercise.

Her total PLG treatment duration was about 306 weeks (5.9 years through March 2022), after which she was transitioned to the commercially available product.

## Discussion

PLGD-1, an ultra-rare, lifelong disease, results in fibrinous lesion accumulation most commonly on mucosal surfaces. Lesion location dependent, PLGD-1 clinical manifestations may lead to significant morbidity or even death. Before the approval of IV PLG for treatment of PLGD-1, many nonspecific interventions were used; most were ineffective or inconsistently effective. Surgical removal of fibrinous lesions provides short-term relief but is often followed by rapid lesion regrowth without adequate substitutive therapy (2). Some case reports suggest the efficacy of topical plasminogen ophthalmic drops in treating patients with LC, with a prospective study which confirmed the efficacy of this treatment modality (17–22).

Management of patients with tracheobronchial involvement is challenging. Treatment with inhaled and systemic corticosteroids in conjunction with prophylactic antibiotics, daily physiotherapy, and inhaled heparin have limited long-term benefits and is associated with complications of chronic steroid therapy (2). FFP, by local or intravenous administration, may be used in the absence of availability of PLG. FFP has had some clinical success, although lack of response, recurrence, and treatment-limiting burden and adverse reactions are reported (9–12). A case report utilizing IV Lys-plasminogen concentrate indicated improvement and resolution of respiratory tract hyperviscous secretions (8, 16, 18).

This case series describes the clinical experiences of three young children and one adult, with severe and life-threatening respiratory complications of PLGD-1, who received replacement therapy with PLG concentrate. This product, which contains Glu-plasminogen (a half-life of 2.2 days compared to Lys-plasminogen with a half-life of 0.8 days in healthy subjects), effectively resolved and prevented lesion recurrence in patients with PLGD-1 and represents a significant improvement in the management of patients with this disorder (7).

The three pediatric cases illustrate the potential life-threatening consequences of respiratory involvement in this disorder and the importance of early disease recognition and effective treatment administration. Lack of effective treatment can result in risk of airway obstruction and mortality and chronic and irreversible sequelae, which is noted in patient 4. PLG replacement therapy provided an immediate increase in plasminogen levels and all patients experienced rapid improvement and resolution of respiratory symptoms. Regression of life-threatening lesions within the respiratory tract and restoration of adequate respiratory function were appreciated on follow-up evaluation. Long-term therapy for up to 306 weeks (about 6 years) was well tolerated and prevented recurrence of respiratory lesions and the development of serious sequelae in all four patients.

Pediatric patients with PLGD with respiratory lesions, especially those with small airways, require close monitoring and rescuscitation capabilities during treatment initiation. Lesions can rapidly detach (slough) after the first dose, potentially causing acute airway obstruction.

Two other previously reported cases highlight the importance of early and effective recognition and treatment (2, 23). A 22-month-old male diagnosed with PLGD-1 at four weeks of age with difficult-to-treat LC and a plasminogen activity level <2% experienced respiratory complications so severe that he suffered cardiopulmonary arrest due to airway obstruction. After resuscitation, he developed a persistent brain injury and required continuous ventilatory and circulatory support. Subsequent administration of PLG led to rapid resolution of the pulmonary lesions and successful extubation, although he was left with persistent brain injury. Another patient, male, age 16 years, experienced chronic mixed obstructive and restrictive lung disease, recurrent pneumonia, and atelectasis for which he was treated with inhaled or systemic corticosteroids, prophylactic antibiotics, daily physiotherapy, and inhaled heparin. He developed severe pseudomonous pneumonia causing a critical reduction in lung capacity and bilateral otitis media with subsequent hearing loss secondary to ligneous deposits in the middle ear. PLG replacement therapy markedly improved his lung function and resolved his hearing loss and abdominal pain. He discontinued treatment with systemic corticosteroids and inhaled heparin, gained weight, and experienced substantial improvement in his quality of life.

The four patients described in this case series support the clinical benefit of replacement therapy with IV PLG to resolve the life-threatening PLGD-1 associated respiratory complications. PLGD-1 associated organ manifestations were improved or resolved with continued treatment. Clinical experience for the use of plasminogen for this rare disease is presently limited and there is insufficient evidence to determine the need for continuous vs. intermittent prophylaxis, except in a sub-set of patients. Patients who present very early in life with multisystem disease, especially those with respiratory lesions, congenital hydrocephalus or persistent/recurrent lesions despite efforts to wean from therapy, will likely need continuous replacement therapy. A clinical research study is currently being conducted to address these knowledge gaps (NCT03797495; Study of Individuals Affected with Hypoplasminogenemia (HISTORY) (14). Early disease recognition and intervention with effective treatment can have life-saving results for patients with PLGD-1.

## Data Availability

The original contributions presented in the study are included in the article/supplementary materials, further inquiries can be directed to the corresponding author.
